# A complement C4–derived glycopeptide is a biomarker for PMM2-CDG

**DOI:** 10.1172/jci.insight.172509

**Published:** 2024-04-08

**Authors:** Kishore Garapati, Rohit Budhraja, Mayank Saraswat, Jinyong Kim, Neha Joshi, Gunveen S. Sachdeva, Anu Jain, Anna N. Ligezka, Silvia Radenkovic, Madan Gopal Ramarajan, Savita Udainiya, Kimiyo Raymond, Miao He, Christina Lam, Austin Larson, Andrew C. Edmondson, Kyriakie Sarafoglou, Nicholas B. Larson, Hudson H. Freeze, Matthew J. Schultz, Tamas Kozicz, Eva Morava, Akhilesh Pandey

**Affiliations:** 1 Department of Laboratory Medicine and Pathology, Mayo Clinic, Rochester, Minnesota, USA; 2 Institute of Bioinformatics, International Technology Park, Bangalore, India; 3 Manipal Academy of Higher Education (MAHE), Manipal, India; 4 Department of Clinical Genomics; 5 Biochemical Genetics Laboratory, Department of Laboratory Medicine and Pathology, Mayo Clinic, Rochester, Minnesota, USA; 6 Department of Pathology and Laboratory Medicine, Children’s Hospital of Philadelphia, Philadelphia, Pennsylvania, USA; 7 Center for Integrative Brain Research, Seattle Children’s Research Institute, Seattle, Washington, USA; 8 Division of Genetic Medicine, Department of Pediatrics, University of Washington School of Medicine, Seattle, Washington, USA; 9 Colorado Children’s Hospital, Denver, Colorado, USA; 10 Division of Human Genetics, Department of Pediatrics, Children’s Hospital of Philadelphia, Philadelphia, Pennsylvania, USA; 11 Division of Pediatric Endocrinology, Department of Pediatrics, University of Minnesota Medical School, Minneapolis, Minnesota, USA; 12 Department of Experimental and Clinical Pharmacology, University of Minnesota School of Pharmacy, Minneapolis, Minnesota, USA; 13 Department of Quantitative Health Sciences, Mayo Clinic, Rochester, Minnesota, USA; 14 Sanford Children’s Health Research Center, Sanford Burnham Prebys Medical Discovery Institute, La Jolla, California, USA; 15 Department of Anatomy, University of Pécs Medical School, Pécs, Hungary; 16 Department of Genomics and Genetic Sciences, Icahn School of Medicine at Mount Sinai Hospital, New York, New York, USA; 17 Center for Individualized Medicine, Mayo Clinic, Rochester, Minnesota, USA

## Abstract

**Background:**

Diagnosis of PMM2-CDG, the most common congenital disorder of glycosylation (CDG), relies on measuring carbohydrate-deficient transferrin (CDT) and genetic testing. CDT tests have false negatives and may normalize with age. Site-specific changes in protein N-glycosylation have not been reported in sera in PMM2-CDG.

**Methods:**

Using multistep mass spectrometry–based N-glycoproteomics, we analyzed sera from 72 individuals to discover and validate glycopeptide alterations. We performed comprehensive tandem mass tag–based discovery experiments in well-characterized patients and controls. Next, we developed a method for rapid profiling of additional samples. Finally, targeted mass spectrometry was used for validation in an independent set of samples in a blinded fashion.

**Results:**

Of the 3,342 N-glycopeptides identified, patients exhibited decrease in complex-type N-glycans and increase in truncated, mannose-rich, and hybrid species. We identified a glycopeptide from complement C4 carrying the glycan Man_5_GlcNAc_2_, which was not detected in controls, in 5 patients with normal CDT results, including 1 after liver transplant and 2 with a known genetic variant associated with mild disease, indicating greater sensitivity than CDT. It was detected by targeted analysis in 2 individuals with variants of uncertain significance in *PMM2*.

**Conclusion:**

Complement C4–derived Man_5_GlcNAc_2_ glycopeptide could be a biomarker for accurate diagnosis and therapeutic monitoring of patients with PMM2-CDG and other CDGs.

**Funding:**

U54NS115198 (Frontiers in Congenital Disorders of Glycosylation: NINDS; NCATS; *Eunice Kennedy Shriver* NICHD; Rare Disorders Consortium Disease Network); K08NS118119 (NINDS); Minnesota Partnership for Biotechnology and Medical Genomics; Rocket Fund; R01DK099551 (NIDDK); Mayo Clinic DERIVE Office; Mayo Clinic Center for Biomedical Discovery; IA/CRC/20/1/600002 (Center for Rare Disease Diagnosis, Research and Training; DBT/Wellcome Trust India Alliance)

## Graphical abstract

**Graphical abstract:**
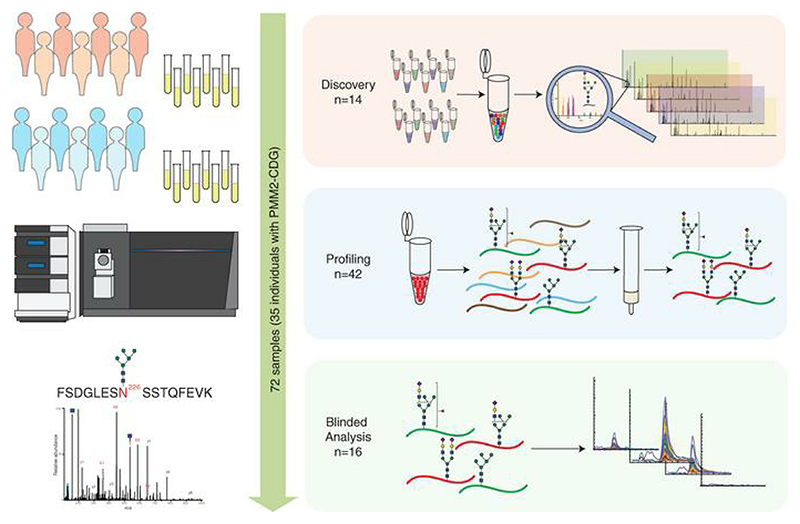

## Introduction

**Figure 1:**
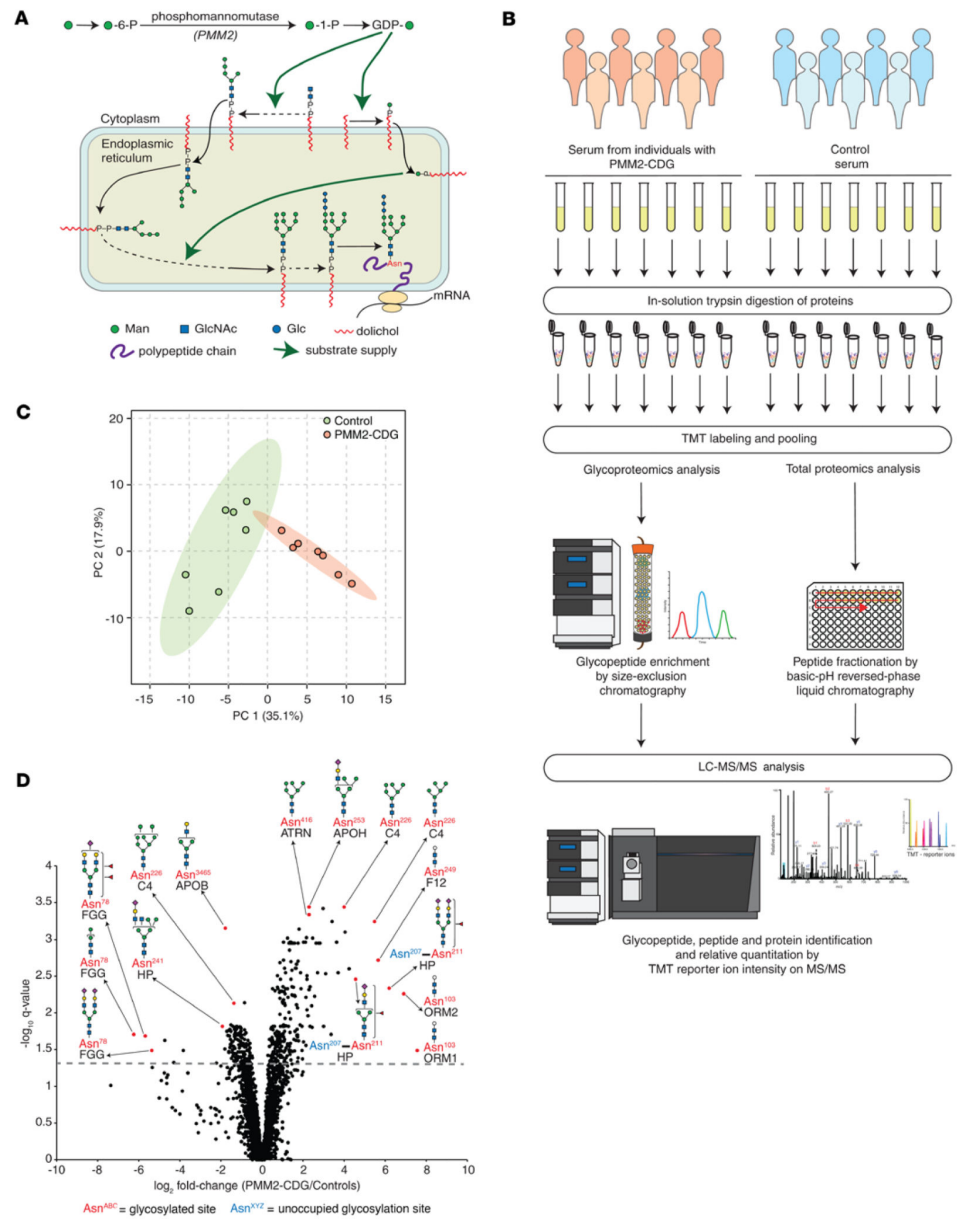
Glycopeptide alterations in PMM2-CDG. (**A**) Overview of protein N-glycosylation and role of phosphomannomutase (encoded by *PMM2*). (**B**) Experimental schematic for TMT labeling–based quantitative glycoproteomics and proteomics. TMT, tandem mass tag. (**C**) Principal component analysis (PCA) plot for TMT-based glycoproteomics data; samples from individuals affected with PMM2-CDG (*n* = 7) and controls (*n* = 7) are represented by circles as indicated. (**D**) Volcano plot showing global glycosylation changes in individuals with PMM2-CDG (*n* = 7) in comparison with controls (*n* = 7); each point represents a glycopeptide with the glycoprotein from which it is derived labeled in black and the amino acid site of glycosylation in red; where the detected glycopeptide backbones contain multiple known glycosylation sites, the nonglycosylated site is labeled in blue; putative structures are shown using symbol nomenclature for glycans (SNFG) and represent glycan composition inferred from mass spectrometry data (63).

Congenital disorders of glycosylation (CDGs) are rare genetic diseases affecting the enzymatic addition of glycans to proteins and lipids. The most common disorder, PMM2-CDG, is caused by a deficiency of phosphomannomutase encoded by the *PMM2* gene, which converts mannose-6-phosphate into mannose-1-phosphate. Pathogenic variants in this gene reduce the amount and availability of glycosylation precursors, such as GDP-mannose primarily for protein N-linked glycosylation (Figure 1A) (1). This autosomal recessive disorder has an estimated prevalence of 1:20,000 to 1:80,000 (2–4). PMM2-CDG symptoms include global developmental delay, ataxia, seizures, and progressive neuropathy, along with endocrine and coagulation abnormalities. Severe infections, liver insufficiency, or cardiomyopathy cause lethality in 20% of patients by age of 6 years (2, 5, 6). PMM2-CDG is often missed in the diagnostic odyssey because of its nonspecific presentation, e.g., hypotonia and feeding difficulties (5). Molecular genetic diagnosis of PMM2-CDG still remains challenging (7). The first hint is often obtained from biochemical analysis of intact serum transferrin by mass spectrometry (MS) or other methods indicating carbohydrate-deficient transferrin (CDT; transferrin molecules lacking 1 or both N-glycan units) (8–11). However, these methods sometimes show normal or borderline abnormal profiles that may normalize upon repeat measurements in both young and older patients (12–15).

Some studies of patients with PMM2-CDG suggest that, in addition to unoccupied glycosylation sites, more subtle changes occur on the N-glycans that are added to a broad array of proteins (16, 17). Although glycoproteomic analysis for characterization of N-glycosylation (i.e., MS-based analysis of intact glycopeptides) is increasingly being applied to study biological problems (18–21), no such analysis of serum samples from patients with PMM2-CDG has yet been published. Thus, we applied our recently described liquid chromatography-tandem mass spectrometry–based (LC-MS/MS-based) N-glycoproteomics methods to study site-specific glycosylation changes in serum proteins in a large cohort of individuals with PMM2-CDG (22, 23). Here, we describe our biomarker discovery efforts progressing from discovery to validation using blinded sample sets. First, we obtained untargeted serum glycoproteomics data from a discovery set of 7 well-phenotyped individuals with PMM2-CDG and 7 controls. We confirmed the presence of unoccupied glycosylation sites in affected individuals and identified a diverse set of glycopeptide signatures that could distinguish individuals with PMM2-CDG from controls. These included glycopeptides from complement C4, alpha-1-acid glycoproteins 1 and 2, coagulation factor XII, and haptoglobin. Second, in the validation stage, we used 2 separate sets of samples from affected individuals for single-shot untargeted analysis followed by targeted analysis by parallel reaction monitoring-mass spectrometry (PRM-MS). We observed that a Man_5_GlcNAc_2_ glycopeptide from complement C4 was detected only in individuals with PMM2-CDG and not in controls. Notably, this glycopeptide was also detected in 5 affected individuals with normal CDT, including 2 individuals with a known genetic variant associated with mild disease. Interestingly, this glycopeptide was also detected in an affected individual from whom the sample was obtained after liver transplantation, even though CDT was negative. Further, in another individual enrolled in a clinical trial, the abundance of this glycopeptide was decreased after 6 months of treatment with the aldose reductase inhibitor epalrestat, though CDT results were unchanged. Finally, this glycopeptide was also detected in 2 affected individuals with abnormal CDT results whose genetic testing showed variants of uncertain significance (VUSs) in *PMM2* gene. These data indicate that this glycopeptide has a greater concordance with the disease status than CDT. This glycopeptide could be deployed for the diagnosis of patients suspected to have PMM2-CDG as well as for monitoring of confirmed patients on treatment in the future.

## Results

### Demographics and clinical features of affected individuals

Thirty-five individuals affected with PMM2-CDG were included in this study; the salient features of this group are summarized in Table 1. The demographic, clinical, and biochemical information of these individuals is shown in detail in Tables 2 and 3, along with severity reported as NPCRS scores (24, 25). All affected individuals had ataxia and global developmental delay. However, 7 individuals were able to walk without support (corresponding to samples 10, 22, 23, 25, 26, 30, and 36; Table 2), and 4 individuals had learning disabilities without intellectual disability (samples 20, 22, 26, and 30). All affected individuals had multisystem disease including peripheral organ involvement, in addition to neurologic involvement. Nine individuals had seizures (samples 6, 7, 11, 13, 18, 19, 27, 28, and 35), and 1 had stroke-like episodes within 6 months of sample collection (sample 1). Two siblings with a shared genotype (*PMM2*, p.R141H in 1 allele and the noncoding variant c.640-23A>G in the second allele) had retinitis pigmentosa and sensorineural hearing loss (samples 27 and 28). However, 10 affected individuals had no signs of neuropathy at the time of investigation (samples 5, 8, 17, 19, 22, 23, 26, 30, 32, and 36). One individual had recurrent deep venous thrombosis of extremities (sample 11). One affected individual was born with a complex cardiac malformation, i.e., transposition of the great arteries (sample 7), and another affected individual had gastrointestinal dysmotility requiring feeding through gastrostomy-jejunostomy tube (sample 12). One affected individual had a severe chest deformity, i.e., pectus carinatum (sample 9), and another had severe scoliosis requiring surgery (sample 16). Twenty-six of the 35 affected individuals had abnormal levels for the activity of N-glycosylated coagulation factors IX, XI, antithrombin, and protein C and endocrine parameters (adrenocorticotropic hormone, thyroid stimulating hormone, insulin-like growth factor binding protein) and normal liver transaminases (Table 3).

One affected individual donated 2 samples collected at different time points (samples 3 and 15). This individual was enrolled in a single-patient investigational new drug (IND) protocol with epalrestat, an aldose reductase inhibitor previously shown to improve phosphomannomutase enzyme activity and glycosylation in vitro (26). Sample 3 was collected from this individual at the age of 6 years at baseline, i.e., before starting treatment with epalrestat, and sample 15 was collected 6 months later on epalrestat treatment. Another affected individual (sample 24) had an unusual course of disease with early progressive liver failure. This individual developed intermittent vomiting, chronic abdominal pain, and recurrent ascites requiring diuretic therapy and repeated paracenteses. Her liver ultrasound showed cirrhosis. Due to end-stage liver disease, hepatic encephalopathy, and esophageal variceal bleeding, she underwent a deceased donor orthotopic liver transplant at the age of 4 years. Although a pretransplantation serum sample was not available, a sample after liver transplantation was obtained as she was enrolled in a CDG natural history study (sample 24) (27).

The genotypes of all affected individuals have been previously reported as pathogenic except 2: p.T118A (corresponding to sample 29) and p.Y229S (sample 36), which are reported to have “uncertain significance” (ClinVar accessions RCV000310325 and RCV000078596.7, respectively) (26, 28–30). Additionally, no genotype-phenotype correlation was noted in the affected individuals except in cases with the genotype p.C241S, which has previously been described as “mild” because of its association with mild ataxia and variable learning difficulties (28). Two individuals with this variant (samples 25 and 26) had comparable clinical phenotypes with normal CDT results. However, 2 other individuals with this variant (samples 20 and 30) had classic abnormal CDT at the adult ages of 21 and 25, respectively (28).

MS of intact transferrin for detecting CDT was performed as part of the standard of care in all 35 affected individuals (31). Transferrin usually contains 2 biantennary sialylated N-glycans (di-oligo transferrin). Patients with PMM2-CDG typically lack 1 or both of them. The ratio of singly glycosylated transferrin (mono-oligo) to di-oligo is no more than 0.06 in unaffected individuals. The ratio of nonglycosylated (a-oligo) to di-oligo transferrin is no more than 0.011 in unaffected individuals. Mono-oligo to di-oligo transferrin ratios in affected individuals ranged from 0.03 (within reference range) to 1.93. A-oligo to di-oligo ratios ranged from 0.002 (within reference range) to 0.66. Importantly, 5 affected individuals with age in the range 6–23 years had fully normal CDT results, even though they all had genetically and/or enzymatically confirmed PMM2-CDG. Two of these individuals (samples 25 and 26) had the variant p.C241S, which has been associated with “mild” disease (28). Two other individuals with CDT results within the reference range (samples 27 and 28) had “severe” NPCRS scores, while the fifth individual (sample 24) donated the sample after liver transplantation.

### Glycoproteomic analysis of serum proteins

We chose 7 individuals (discovery set: samples 1 to 7, Table 3) with PMM2-CDG along with 7 age- and sex-matched controls for an in-depth analysis of their serum glyco-proteome to identify glycopeptide biomarkers in PMM2-CDG. We first confirmed CDT results by MS-based protein-level measurements with TMT-based relative quantitation: peptides from transferrin with unoccupied N-glycosylation sites were increased in PMM2-CDG (Figure 1B and Supplemental Figure 1A; supplemental material available online with this article; https://doi.org/10.1172/jci.insight.172509DS1). We also identified increased abundance of peptides with unoccupied N-glycosylation sites derived from several other serum proteins in PMM2-CDG (Supplemental Figure 1B). For glycoproteomics analysis, serum-derived peptides were multiplexed with tandem mass tags (TMTs) for relative quantitation followed by N-glycopeptide enrichment by size-exclusion chromatography (SEC) and LC-MS/MS analysis (32, 33) (Figure 1B). A total of 3,342 glycopeptides were identified from 284 glycoproteins, and their distribution by class of glycan is shown in Supplemental Figure 2, A–C. Alpha-1-acid glycoproteins 1 and 2 contributed the largest number of glycopeptides, 297, for any protein. The most frequently identified glycans on glycopeptides were the sialylated biantennary glycans Hex_5_HexNAc_4_NeuAc_2_ (352 glycopeptides) and Hex_5_HexNAc_4_NeuAc_1_ (230 glycopeptides) (Supplemental Table 1). Principal component analysis (PCA) performed using TMT-based relative abundance values of glycopeptides clustered PMM2-CDG and control samples separately (Figure 1C).

### Site-specific alterations in N-glycosylation of serum proteins

**Figure 2:**
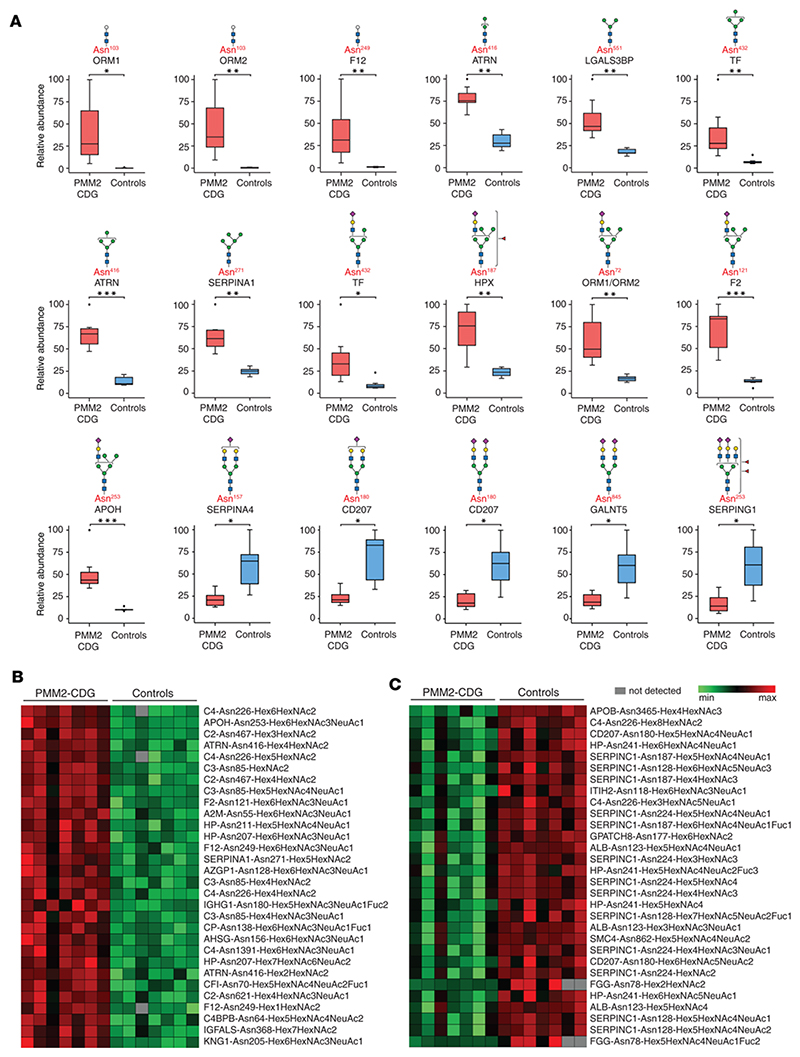
Site-specific alterations in glycopeptide abundance from TMT-based experiments in the discovery set. (**A**) Box plots representing levels of significantly altered glycopeptides; the glycoprotein from which the glycopeptide is derived is labeled in black and the amino acid site of glycosylation in red; putative structures are shown using SNFG and represent glycan composition inferred from mass spectrometry data (63). The box plots depict minimum and maximum values (whiskers), upper and lower quartiles, and median. The length of the box represents the interquartile range. *=*q* < 0.05, **=*q* < 0.01, ***=*q* < 0.001. (**B**) Heatmap showing the most significantly increased (*q* < 0.05) glycopeptides in PMM2-CDG. (**C**) Heatmap showing the most significantly decreased (*q* < 0.05) glycopeptides in PMM2-CDG; glycopeptides are represented by protein followed by the amino acid site of glycosylation and glycan composition. The *q* values in **A**–**C** were calculated by *t* test with multiple testing using Benjamini-Hochberg procedure. PMM2-CDG (*n* = 7), controls (*n* = 7). Hex, hexose; HexNAc, N-acetylglucosamine; NeuAc, N-acetylneuraminic acid; Fuc, fucose.

We found significant glycosylation changes (*q* < 0.05) in 371 unique N-glycopeptides from 85 glycoproteins (Figure 1D and Supplemental Figure 3). A total of 136 glycopeptides from 39 proteins were elevated with an average fold-change (PMM2-CDG/controls) of ≥2-fold (*q* < 0.05). A total of 14 of these glycopeptides bore paucimannose (3 or fewer hexose residues) or chitobiose-core glycan composition. Notably, glycopeptides from 3 proteins, alpha-1-acid glycoprotein 1 (ORM1, glycopeptide containing amino acid glycosylation site Asn^103^), alpha-1-acid glycoprotein 2 (ORM2, Asn^103^), and coagulation factor XII (F12, Asn^249^) bearing the truncated glycan, Hex_1_HexNAc_2_, were found to be elevated ≥50-fold (average fold-change, PMM2-CDG/controls), with undetectable levels in several controls. Glycopeptides bearing the tetrasaccharide, Hex_2_HexNAc_2_ from attractin (ATRN, Asn^416^), complement C3 (C3, Asn^85^), immunoglobulin heavy constant mu chain (IGHM, Asn^46^), and F12 (Asn^249^) were also high in PMM2-CDG. Glycopeptides bearing the paucimannose glycan Hex_3_HexNAc_2_ from F12 (Asn^249^) and galectin-3-binding protein (LGALS3BP, Asn^551^) were also elevated (Figure 2A and Supplemental Table 1).

Oligomannose glycopeptides containing 6 or fewer mannose residues from several proteins were elevated, including transferrin (Asn^432^: Hex_4_HexNAc_2_), ATRN (Asn^416^: Hex_4_HexNAc_2_), and alpha-1-antitrypsin (SERPINA1, Asn^271^: Hex_5_HexNAc_2_) (Figure 2A). A number of hybrid glycopeptides were increased in affected individuals. The glycopeptide from transferrin (Asn^432^) with glycan composition Hex_5_HexNAc-_3_NeuAc_1_ was elevated in PMM2-CDG. Glycopeptides from several glycoproteins with the hybrid glycan composition Hex_6_HexNAc_3_NeuAc_1_Fuc_1_, corresponding to a fucosylated hybrid glycan, were elevated: ceruloplasmin (Asn^138^), immunoglobulin heavy chain constant gamma 1 (IGHG1, Asn^180^), and IGHM (Asn^209^) (Figure 2A). Notably, the levels of hybrid glycopeptides with the glycan composition Hex_6_HexNAc_3_NeuAc_1_ were increased on 25 glycosylation sites derived from 19 proteins, including ORM1/ORM2 (Asn^72^), prothrombin (F2, Asn^121^), and beta-2-glycoprotein 1 (Asn^253^) (Figure 2A and Supplemental Table 1).

We noted that 87 glycopeptides derived from 24 proteins had decreased abundance (average fold-change ≤ 0.5, *q* < 0.05) in PMM2-CDG. A majority of these were of the hybrid/complex type, with high-mannose glycopeptides accounting for a small minority. Complex mono- and di-sialylated biantennary glycans accounted for 20 of these glycopeptides derived from several proteins, including kallistatin (SERPINA4, Asn^157^), CD207 (Asn^180^), and N-acetylgalactosaminyltransferase 5 (Asn^845^). Complex di- and tri-sialylated triantennary glycans accounted for an additional 13 glycopeptides decreased in abundance, mainly from plasma protease C1 inhibitor (SERPING1, Asn^253^), clusterin (Asn^86^), and transferrin (Asn^630^) (Figure 2A and Supplemental Table 1). Box plots representing significantly altered glycopeptides between PMM2-CDG and control groups are shown in Figure 2A, and the most significantly elevated and decreased glycopeptides (average fold-change, PMM2-CDG/controls, *q* < 0.05), are shown in Figure 2, B and C. Among the significantly decreased glycopeptides shown in Figure 2C, 3 glycopeptides were from albumin, with glycosylation at the noncanonical motif Asn^123^-Glu-Cys. We discovered this N-linked glycosylation site in albumin in a recent exploratory study (34), and the current finding indicates that glycosylation at this site, which we believe to be novel, is also affected in PMM2-CDG.

### Changes in glycan microheterogeneity

**Figure 3:**
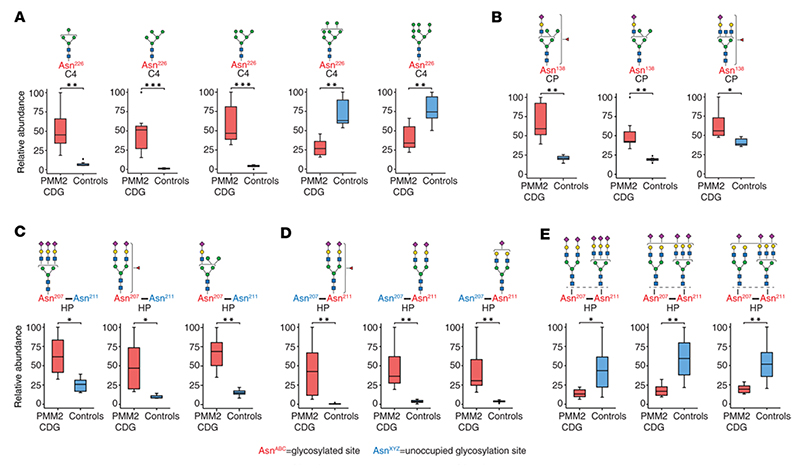
Microheterogeneity in glycosylation for selected proteins from TMT-based experiments in the discovery set. Box plots showing relative abundance of glycopeptides bearing different glycans at the same site in selected proteins (**A**) complement C4 Asn^226^ and (**B**) ceruloplasmin Asn^138^. (**C**) Representative haptoglobin-derived glycopeptides containing Asn^207^ and Asn^211^ but with glycan occupancy only at Asn^207^, with Asn^211^ unoccupied. (**D**) Representative haptoglobin-derived glycopeptides containing Asn^207^ and Asn^211^ but with glycan only at Asn^211^, with Asn^207^ unoccupied. (**E**) Representative haptoglobin-derived glycopeptides containing Asn^207^ and Asn^211^ with glycosylation at both Asn^207^ and Asn^211^; putative structures are shown using SNFG and represent total composition of glycan(s) inferred from mass spectrometry data (63). The box plots depict minimum and maximum values (whiskers), upper and lower quartiles, and median. The length of the box represents the interquartile range. PMM2-CDG (*n* = 7), controls (*n* = 7); *=*q* < 0.05, **=*q* < 0.01, ***=*q* < 0.001; the *q* values in **A**–**E** were calculated by *t* test with multiple testing using Benjamini-Hochberg procedure.

We observed interesting patterns of change in the overall micro-heterogeneity at some glycosylation sites in PMM2-CDG. Several oligomannose glycopeptides at Asn^226^ of complement C4 showed significant changes: Man_4_GlcNAc_2_, Man_5_GlcNAc_2_, and Man_6_GlcNAc_2_ glycopeptides were elevated while Man_7_GlcNAc_2_, Man_8_GlcNAc_2_, and Man_9_GlcNAc_2_ glycopeptides were reduced in PMM2-CDG. Specifically, Man_5_GlcNAc_2_ and Man_6_GlcNAc_2_ glycopeptides from C4 (Asn^226^) were elevated 45- and 16-fold, respectively, in PMM2-CDG (average fold-change PMM2-CDG/controls; *q* = 0.0006 and 0.0004, respectively) (Figure 3A and Supplemental Table 1). Interestingly, data from the protein-level measurements showed that complement proteins C4A and C4B (both of which share the tryptic peptide sequence that contains this glycosylation site) were not significantly different in their abundance in PMM2-CDG at the protein level (average fold-change PMM2-CDG/controls of 1.2 and 1.1, respectively, with *q* = 0.41 and 0.52, respectively). However, the nonglycosylated peptide containing the glycosylation site Asn^226^ was elevated 6-fold in PMM2-CDG compared with controls (*q* = 0.0099) (Supplemental Figure 1). This suggests that there are 2 changes occurring at this site in PMM2-CDG: the first, an overall decrease in glycosylation site occupancy; and second, where the glycosylation site is occupied, Man_5_GlcNAc_2_ and Man_6_GlcNAc_2_ glycans are increased and Man_8_GlcNAc_2_ or Man_9_GlcNAc_2_ glycans are decreased.

Another interesting pattern was observed at Asn^138^ of ceruloplasmin where hybrid glycans with the composition Hex_6_HexNAc_3_NeuAc_1_Fuc_1_ and Hex_6_HexNAc_3_NeuAc_1_ and the complex monosialylated glycan Hex_5_HexNAc_5_NeuAc_1_Fuc_1_ were significantly increased in PMM2-CDG (Figure 3B). The peptide from ceruloplasmin with nonglycosylated Asn^138^, however, was elevated 5-fold in PMM2-CDG (*q* = 0.0096) while the protein level was relatively unchanged (average fold-change PMM2-CDG/controls of 1.05, *q* = 0.69) (Supplemental Figure 1). This suggests a decrease in glycosylation at this site along with a significant increase in hybrid glycans where the site is glycosylated.

### Hypoglycosylation of specific sites on haptoglobin

Because glycosylation changes in haptoglobin have previously been described in the context of PMM2-CDG (35, 36), we took a closer look at this glycoprotein. Haptoglobin-derived tryptic peptide NLFLN^207^HSEN^211^ATAK contains 2 known glycosylation sites. The fully glycosylated peptide (both sites occupied) was decreased while peptides glycosylated on a single site were increased in PMM2-CDG (Figure 3, C–E). To our knowledge, this finding has not been previously described for any haptoglobin-derived glycopeptide with multiple glycosylation sites in PMM2-CDG. We were able to do this because of the peptide- and glycosylation site-specific analysis that is now possible with recent advances in MS and database searching capabilities (22, 23, 37). These findings indicate substantial hypoglycosylation of these sites in haptoglobin in PMM2-CDG.

### Streamlined glycoproteomic profiling

**Figure 4:**
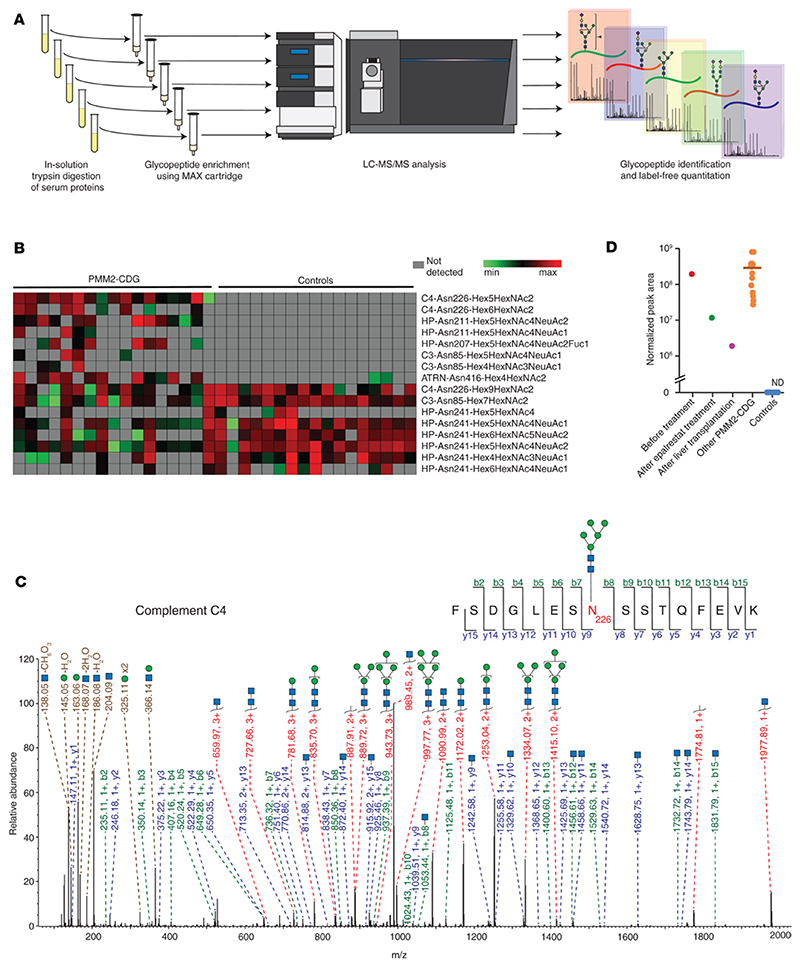
Profiling N-glycosylation using MAX cartridge–based enrichment and single-shot MS analysis. (**A**) Strategy for MAX-based enrichment and analysis. (**B**) Heatmap showing glycopeptides identified by label-free analysis using discovery methods; glycopeptides are represented by protein followed by the amino acid site of glycosylation and glycan composition. PMM2-CDG (*n* = 17), controls (*n* = 17). Hex, hexose; HexNAc, N-acetylglucosamine; NeuAc, N-acetylneuraminic acid. Fuc, fucose. (**C**) Annotated MS/MS spectrum for the Man_5_GlcNAc_2_ glycopeptide from complement C4 at site Asn^226^; putative structures are shown using SNFG and represent glycan composition inferred from MS data (63). (**D**) Dot plot representing normalized peak intensity values for the Man_5_GlcNAc_2_ glycopeptide from complement C4 at site Asn^226^ in PMM2-CDG and control samples. Each dot represents abundance in a sample from an individual, and the horizontal bar represents the median; special cases are labeled and shown separately as follows: “Before treatment”: sample 3 (*n* = 1) was donated by an individual affected with PMM2-CDG before treatment with epalrestat; “After epalrestat treatment”: sample 15 (*n* = 1) was donated by the same individual at 6 months of therapy with epalrestat; “After liver transplantation”: sample 24 (*n* = 1) was donated by an affected individual after liver transplantation; other PMM2-CDG (*n* = 15); controls (*n* = 17). ND, not detected.

Although our multiplexed workflow using TMT labeling followed by SEC is excellent for discovery studies, it is not practical for analysis of clinical samples as it requires long hands-on time for sample preparation in addition to longer MS analysis time. To address this, we focused on an independent set of 17 samples from individuals with PMM2-CDG (samples 8 to 24) and 17 controls using a more streamlined method in which glycopeptides were enriched using mixed-mode anion exchange (MAX) cartridges (Figure 4A) and each sample analyzed separately by LC-MS/MS in a single-shot run (Table 3, profiling set).

Figure 4B shows a heatmap of the most prominent glycosylation changes in PMM2-CDG. The only glycopeptide that was found in all 17 samples but absent in all controls was the glycopeptide with amino acid sequence and glycan composition FSDGLESN^226^(Man_5_GlcNAc_2_)SSTQFEVK derived from complement C4. Several other glycopeptides were found in many, but not all, samples from individuals with PMM2-CDG and absent from all controls. Haptoglobin-derived glycopeptides with the peptide sequence NLFLN^207^HSEN^211^ATAK, which contain 2 glycosylation sites but are only glycosylated at 1 of them, were only detected in samples from affected individuals; for example, glycopeptides with composition Hex_5_Hex-NAc_4_NeuAc_2_ at Asn^211^ and Hex_5_HexNAc_4_NeuAc_2_Fuc_1_ at Asn^207^ were found in 12 and 8 affected individuals, respectively, but in none of the controls. The glycopeptide from ATRN with Man_4_GlcNAc_2_ at Asn^416^ was found in samples from 11 affected individuals and with low intensity in only 2 controls. Many complement C4–derived glycopeptides observed to be elevated in the initial discovery set were also identified in this profiling set. Similar to TMT-based discovery data, the profiling set revealed a differential abundance of high-mannose-containing glycopeptides from C4 (Asn^226^) in individuals with PMM2-CDG. The glycopeptides from C4 (Asn^226^) bearing Man_6_GlcNAc_2_ and fewer mannose residues were only found in individuals with PMM2-CDG; on the other hand, in control samples, only glycopeptides with the composition Man_7_GlcNAc_2_, Man_8_GlcNAc_2_, and Man_9_GlcNAc_2_ were detected at this site. Man_6_GlcNAc_2_ glycopeptide was identified in 8 affected individuals but not in any of the control samples. The peak area for glycopeptides bearing Man_7-9_GlcNAc_2_ was lower in all individuals affected with PMM2-CDG. A representative MS/MS spectrum from the sample of an affected individual for the Man_5_GlcNAc_2_ glycan-bearing glycopeptide derived from complement C4 (Asn^226^) is shown in Figure 4C. By the streamlined profiling method using MAX-based enrichment, we did not detect some of the paucimannose glycopeptides that were identified to be elevated in affected individuals in the TMT-based discovery experiment. This could be due to single-shot LC-MS/MS runs of MAX-enriched fractions not being as comprehensive as the analysis of multiple fractions in the TMT-based discovery experiment. Another reason could be the differences in the basis of the enrichment methods.The downregulated glycopeptides common to the discovery and profiling sets also included a Man_7_GlcNAc_2_ glycopeptide on Asn^85^ site of complement C3, which was identified in 15 out of 17 affected individuals and all 17 controls. The peak area of this glycopeptide was lower in all individuals with PMM2-CDG as compared with controls. Eight other glycopeptides that were decreased in samples from individuals with PMM2-CDG were complex and hybrid glycopeptides containing site Asn^241^ of haptoglobin, which is concordant with changes observed at this site in the TMT-based experiment.

This cohort included an affected individual under a single-patient IND protocol who was sampled twice. Sample 15 was collected from this individual at 6 months of treatment with the aldose reductase inhibitor epalrestat, which was being tested to improve phosphomannomutase (PMM2) enzyme activity. We had access to an archived sample from this individual at baseline, i.e., before treatment with epalrestat (sample 3), which was also analyzed using this streamlined method. The sample drawn after treatment showed a substantially lower peak area for the Man_5_GlcNAc_2_ bearing glycopeptide derived from C4 (Asn^226^) as compared with the sample drawn before treatment (ratio of normalized peak areas, at 6 months of treatment to baseline, 0.06). Another interesting case involved sample 24, which was donated by a different individual after liver transplantation. Although a corresponding pretransplantation sample was unavailable for this individual, the peak area for this glycopeptide was markedly lower than in all other samples from individuals with PMM2-CDG (ratio of normalized peak areas, sample 24 to average of all other affected individuals, 0.007) (Figure 4D).

### Development of a targeted MS assay

**Figure 5:**
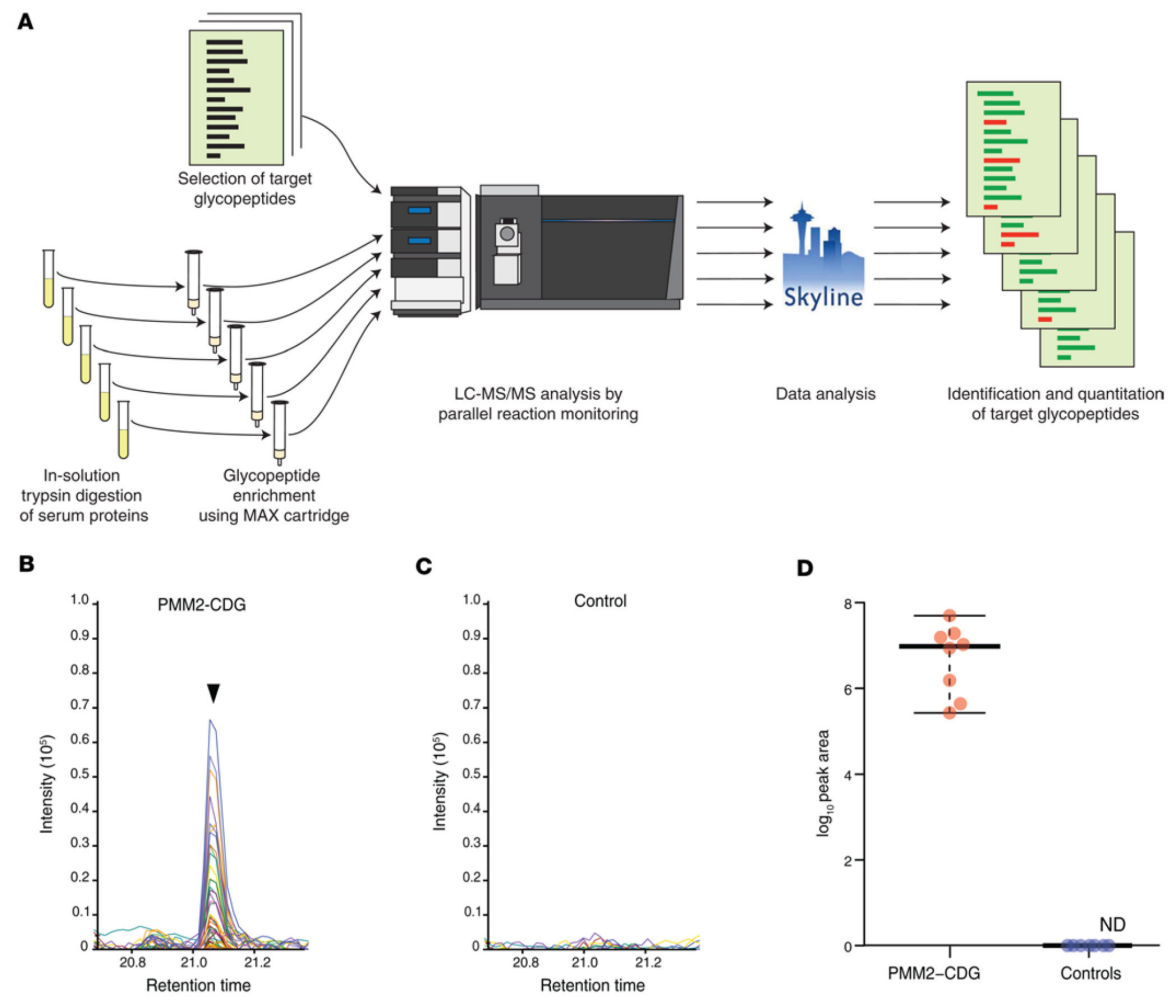
Detection of Man_5_GlcNAc_2_ glycopeptide from complement C4 by targeted PRM assay. (**A**) Strategy for MAX-based enrichment and targeted analysis of glycopeptides. (**B**) Representative extracted ion chromatogram showing abundance of Man_5_GlcNAc_2_ glycopeptide from complement C4 at Asn^226^ in an individual with PMM2-CDG. (**C**) Representative extracted ion chromatogram at the same retention time in a control sample. (**D**) Dot plot showing abundance of the Man_5_GlcNAc_2_ glycopeptide from complement C4 at Asn^226^ in PMM2-CDG (*n* = 8) and control (*n* = 8) samples used for development of PRM assay. The dots represent abundance in each sample, and the horizontal bars represent, from top to bottom, the maximum value, median, and minimum value, respectively.

Encouraged by the results above, we developed a targeted method where MAX-enriched samples were analyzed by PRM for selected candidate glycopeptides. This method is more suitable for analysis in a clinical setting due to lower MS analysis time as well as enhanced sensitivity (Figure 5A). Four glycopeptide candidates were selected for targeted analysis based on the TMT-based discovery experiment and profiling experiments with MAX-enriched glycopeptides. The targeted glycopeptides are EN^103^(Hex_1_HexNAc_2_)GTISR (alpha-1-acid glycoprotein 1, ORM1), EN^103^(Hex_1_HexNAc_2_)GTVSR (alpha-1-acid glycoprotein 2, ORM2), N^249^(Hex_1_HexNAc_2_)VTAEQAR (coagulation factor XII, F12), and FSDGLESN^226^(Man_5_HexNAc_2_)SSTQFEVK (complement C4). The PRM assay for the selected peptides was first tested on 8 samples from individuals with a known diagnosis of PMM2-CDG and 8 controls (Table 3, samples 21 to 28). This included a subset of samples in which profiling experiments were already carried out, along with an additional 4 samples from individuals with PMM2-CDG who were reported to have control-like profiles based on their CDT results. The 3 target glycopeptides from ORM1, ORM2, and F12 bearing the paucimannose glycan Hex_1_HexNAc_2_ were not detected in any of the samples. However, the target glycopeptide FSDGLESN^226^(Man_5_HexNAc_2_)SSTQFEVK from C4 was detected in all 8 samples from affected individuals but in none of the controls. This target was detected as a doubly charged precursor ion with *m/z* 1,496.1216 at a retention time of 21 minutes. Importantly, this glycopeptide was detected in 5 individuals with PMM2-CDG who had CDT results within the reference range. A representative targeted LC-MS/MS–extracted chromatogram of the target glycopeptide detected in the sample from an affected individual (sample 24) is shown in Figure 5B, and an extracted chromatogram from a control sample at the same retention time is shown in Figure 5C. Levels of this target glycopeptide in samples from affected individuals and controls that were used for testing the targeted assay are shown in Figure 5D.

### PRM assay successfully identifies confirmed cases of PMM2-CDG as well as affected individuals with VUSs

**Figure 6:**
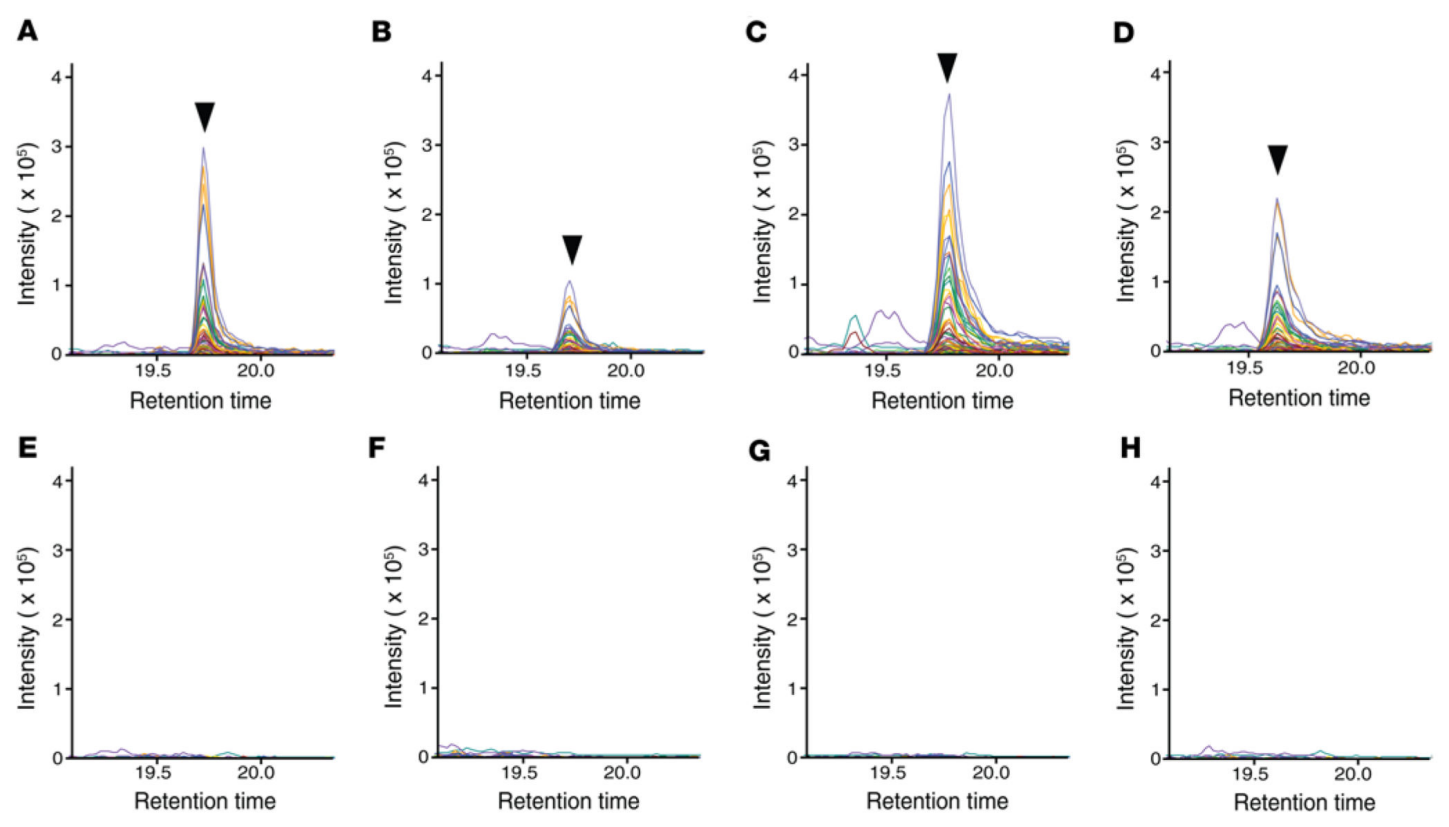
Detection of Man_5_GlcNAc_2_ glycopeptide from complement C4 at Asn^226^ by blinded analysis correctly identifies individuals with PMM2-CDG. Representative Skyline plots showing glycopeptide fragment peaks from selected samples in the blinded set. Targeted analysis was performed on 16 samples by PRM by operators blinded to the identity of the samples. (**A**–**D**) Samples identified to be from individuals with PMM2-CDG. (**E**–**H**) Samples identified to be from controls.

Blinded analysis of 16 samples (PMM2-CDG or control) was performed using this PRM assay. The assay was 100% accurate in assigning individuals to their respective groups based on the targeted detection of the glycopeptide FSDGLESN^226^(Man_5_GlcNAc_2_)SSTQFEVK. This glycopeptide was not detected in any of the control samples. Representative LC-MS/MS–extracted chromatograms for affected individuals are shown in Figure 6, A–D, and extracted chromatograms for controls at the same retention time are shown in Figure 6, E–H. Most importantly, this glycopeptide was also detected in 2 individuals who had VUSs in *trans* with the common variant R141H in *PMM2*: samples 29 (p.T118A, “Uncertain significance,” ClinVar accession RCV000310325) and 36 (p.Y229S, “Uncertain significance,” ClinVar accession RCV000078596.7) (Table 3). Next, to assess if there is any correlation between levels of the complement C4–derived Man_5_ glycopeptide at site Asn^226^ and clinical severity, we analyzed the levels of this glycopeptide along with the NPCRS scores for each affected individual using Kendall’s Tau rank correlation test. We did not observe any correlation between any of the aggregate or component NPCRS scores and the glycopeptide biomarker (Supplemental Tables 3 and 4, respectively).

## Discussion

CDT measured by intact mass analysis of transferrin glycosylation isoforms is the current standard of care in clinical diagnosis of CDG (13), though other methods have also been used (8–11). False negatives are known to occur in several types of CDG, including PMM2-CDG (12). Rare transferrin polymorphisms can also complicate interpretation of CDT assays (38, 39). Other glycoproteins, such as coagulation factors and hormones, have been studied in a similar fashion as transferrin and are sometimes included in CDG phenotyping (13, 40–43). Another diagnostic approach is MS analysis of N-glycans enzymatically released from total serum (44), although this method does not indicate glycosylation site occupancy or identify the proteins with changes in glycosylation (7). Additionally, the measurement of N-glycans released from serum is actually a composite of glycans derived from many proteins. The resulting loss of information on glycosylation of individual proteins reduces the discriminatory capacity of glycomic analyses.

Recent advances in sample preparation, MS, and software development overcome these limitations (45–47), allowing analysis of thousands of glycopeptides to identify putative glycan structure based on composition and known biosynthetic pathways, as well as the protein-specific glycosylation sites at which the glycan is attached (37, 48, 49). Determining definitive glycan structures requires further analysis (50, 51). These glycoproteomics approaches have already begun to reveal global alterations in cancer, infection, and immune dysfunction (18–21) but have not yet been widely applied to CDG. We have developed methods for unbiased enrichment and deep profiling of intact N-glycopeptides by LC-MS/MS and previously applied them to study cellular models of CDG (22, 23, 26, 52, 53).

### Glycoproteomics reveals patterns of altered protein glycosylation in PMM2-CDG

We found a remarkable increase in truncated glycans on many N-glycosylation sites in the serum proteome of PMM2-CDG individuals as compared with controls, where mature glycans were more abundant. The increase of paucimannose glycopeptides on several proteins with a concomitant reduction in Man_8_GlcNAc_2_ and Man_9_Glc-NAc_2_ bearing glycopeptides suggests aberrant N-glycan processing. The β chain of haptoglobin, which contains 4 N-glycosylation sites, was previously described as altered in CDG (35, 36). We found that a tryptic glycopeptide from haptoglobin containing 2 closely spaced N-glycosylation sites (Asn^207^ and Asn^211^) is usually glycosylated at both sites in control individuals. In PMM2-CDG, a lack of glycosylation on one of these sites was noted, which might indicate frequent skipping of glycosylation at closely spaced acceptor sites on a polypeptide sequence (54, 55) and may also be applicable to other proteins in CDG.

### Man_5_GlcNAc_2_ glycopeptide from complement C4 as a biomarker for reliable diagnosis

Finding better biomarkers in PMM2-CDG is a significant unmet need. CDT and phosphomannomutase enzyme activity are sometimes unreliable, complicating diagnosis especially in cases where genetic testing shows VUSs. The Man_5_GlcNAc_2_ glycopeptide derived from complement C4 at site Asn^226^ was detected in all 35 tested individuals with PMM2-CDG, including 5 who had normal CDT results. Specifically, 2 of these 5 individuals carried the “mild” genetic variant (p.C241S) and were scored as clinically mild by the NPCRS but were classified along with other affected individuals based on detection of this glycopeptide. Notably, this marker was also detected in 2 phenotypically affected individuals with VUSs. Our results combined with their clinical course and biochemical abnormalities suggest these variants to be likely pathogenic.

To our knowledge, the cohort of affected individuals included in this study is the largest group with this disorder studied by glycoproteomic methods. However, this biomarker study is still limited by sample size considerations inherent to studying ultra-rare genetic disorders. Another limitation of this study is that this biomarker will need to be tested for its potential applicability in other CDGs. This glycopeptide biomarker after additional validation could be considered for deployment for clinical testing, as well as a part of studying the natural history of PMM2-CDG. Lower levels of this marker detected in an affected individual on treatment with epalrestat and another individual after liver transplant indicate that it could be tested as a potential measure of outcomes in future clinical trials in PMM2-CDG. We also discovered additional glycopeptides and peptides with unoccupied glycosylation sites from abundant plasma proteins that show coordinate changes in samples from individuals with PMM2-CDG. These glycopeptides can be tested as biomarkers in the future using other methods (e.g., targeted methods with heavy-labeled glycopeptides as internal standards) or be incorporated as part of potential panels of glycopeptide biomarkers. To conclude, glycoproteomic profiling for discovery followed by streamlined profiling and validation experiments, enabled discovery of Man_5_GlcNAc_2_ glycopeptide from C4 at site Asn^226^ as a biomarker in PMM2-CDG.

## Methods

### Sex as a biological variable

Our study included both male and female individuals. Affected individuals and control groups were matched for sex.

### Affected individuals

Thirty-six samples from 35 affected individuals (22 male and 13 female, age range = 1–36 years, median age = 10 years) with a molecularly confirmed diagnosis of PMM2-CDG were included in the study. All individuals are enrolled in the Frontiers in Congenital Disorders of Glycosylation Consortium (FCDGC) natural history study upon written informed consent (IRB: 16-004682 and IRB: 19-005187; https://clinicaltrials.gov/ct2/show/NCT04199000?cond=CDG&draw=2&rank=4). Data were collected prospectively and retrospective data was available for most affected individuals as well, as they have been previously followed up by standard of care. Some of the patients were previously reported in Ligezka et al. (26). The demographic, clinical, and genetic information of the affected individuals is presented in Tables 2 and 3.

### Drug administration

As part of a single-patient IND protocol, epalrestat was administered orally to an affected individual (corresponding to samples 3 and 15), 3 times per day (tid) before meals in a divided dose (0.8 mg/kg/d; 5 mg tid) (Ono Pharmaceuticals) between day 1 and day 180 of the study (IRB: 19-010017; IND 145262; Protocol PMM2-CDG001/A) (26).

### Controls

Thirty-six control serum samples were used from unaffected individuals, between the ages of 1–36 (median 10 years) (22 male and 14 female), who donated deidentified residual samples (IRB: 21-012890) (Supplemental Table 2).

### Data extraction

Clinical data were collected and extracted from the electronic clinical files and electronic laboratory data system. The NPCRS was used to assess disease severity. The NPCRS rating was performed in every affected individual at the time of the laboratory sample collection. The NPCRS is a clinical tool developed to determine the clinical severity and disease progression of individuals affected with CDG in an objective way and is validated for all age groups. Depending on the NPCRS score, cases are scaled in a mild (0–14 points), moderate (15–25 points), or severe (>26 points) category (24–26). CDT analysis was performed by MS analysis of intact transferrin. Mono-oligo/di-oligo and A-oligo/di-oligo ratios of glycosylated transferrin were collected for each affected individual (31, 56).

### Serum protein digestion

Bicinchoninic acid protein assay (Pierce, Thermo Fisher Scientific) was used to determine protein concentration of serum samples. Equal amount of total protein from each serum sample was vacuum-dried and reconstituted in 8 M urea in 50 mM triethylammonium bicarbonate (TEAB), pH 8.5. Proteins were reduced by incubation with dithiothreitol at a final concentration of 10 mM (MilliporeSigma) at 37°C for 45 minutes. Samples were then cooled to room temperature and alkylated by incubation with 40 mM iodoacetamide (MilliporeSigma) for 15 minutes in the dark at room temperature. The samples were diluted with 50 mM TEAB to a final urea concentration of less than 1 M, and digestion was carried out with trypsin (Worthington, TPCK treated) at a ratio of 1:20 (trypsin/total protein) at 37°C overnight.

### TMT labeling of peptides and glycopeptide enrichment using SEC

Peptides derived from serum samples of affected individuals (*n* = 7) and controls (*n* = 7) were labeled with TMT (TMTPro, Thermo Fisher Scientific) reagents as per the manufacturer’s instructions. After a label check, samples were pooled and the sample was split into 2 aliquots for glycoproteomics and total proteomics experiments. About 90% of dried peptides were resuspended in 100 μL of 0.1% formic acid and loaded onto Superdex peptide 10/300 column (GE Healthcare, now Cytiva) for SEC. Isocratic flow with 0.1% formic acid was used, and 48 fractions were collected over a run time of 130 minutes. Early fractions from SEC collected over 55 minutes starting at 40 minutes after injection were selected based on the UV profile (214 nm, 220 nm) and used for LC-MS/MS. The remaining aliquot (about 10%) of total peptides was cleaned up by C18 TopTips (Glygen) and fractionated by basic reversed phase liquid chromatography (bRPLC) on a C18 column (4.6 × 100 mm column) using an UltiMate 3000 UHPLC System (Thermo Fisher Scientific) for the total proteomics experiment. We used 5 mM ammonium formate in water, pH 9, and 5 mM ammonium formate in 90% acetonitrile, pH 9, as solvent A and B, respectively. Ninety-six fractions collected over 120 minutes were concatenated into 12 fractions. These fractions were dried down in a speed vacuum system and resuspended in 0.1% formic acid for LC-MS/MS analysis.

### Enrichment of glycopeptides using MAX cartridges

For analysis of the profiling and blinded sets, glycopeptides were enriched from peptide samples using OASIS MAX cartridges (Waters) (57). The MAX cartridges were conditioned with acetonitrile (ACN) thrice, then thrice with 100 mM triethylammonium acetate buffer, followed by thrice with water, and finally thrice with 95% ACN/1% trifluoroacetic acid (TFA). Dried tryptic peptides from sera of affected individuals and controls were reconstituted in 95% ACN/1% TFA. Equal peptide amounts (400 μg) from each sample were used for loading thrice onto the cartridges. The cartridge was then washed thrice with 95% ACN/1% TFA. Glycopeptides were eluted with 50% ACN/0.1% TFA and dried down. The enriched glycopeptides from 18 affected individuals (sample 3 and samples 8 to 24) and 17 matched control serum samples were used for validation experiments using single-run nontargeted analysis. For development and testing of the targeted glycopeptide assay, 16 samples from affected individuals (samples 21 to 36) and 16 matched control samples enriched using MAX cartridges were analyzed using PRM-based targeted analysis.

### LC-MS/MS analysis for discovery and profiling experiments

Previously published LC-MS/MS parameters (22, 23, 52) were used with some modifications. Briefly, for the TMT-based experiments, 11 early fractions from SEC and 12 concatenated fractions from bRPLC were analyzed by Orbitrap Exploris 480 mass spectrometer (Thermo Fisher Scientific) for glycoproteomics and proteomics, respectively. Peptides were separated by liquid chromatography on an EASY-Spray column (75 μm × 50 cm, PepMap RSLC C18, Thermo Fisher Scientific) packed with 2 μm C18 particles, maintained at 50°C. We used 0.1% formic acid in water (solvent A) and 0.1% formic acid in acetonitrile (solvent B) as solvents. Peptides were trapped on a trap column (100 mm × 2 cm, Acclaim PepMap100 Nano-Trap, Thermo Fisher Scientific) at a flow rate of 20 μL/min. LC separation was performed at a flow rate of 300 nL/min, and the following gradient was used: equilibration at 3% solvent B from 0 to 4 minutes, 3% to 10% sol B from 4 to 10 minutes, 10% to 35% sol B from 10.1 to 125 minutes, 35% to 80% sol B from 125 to 145 minutes, followed by equilibration for next run at 5% sol B for 5 minutes. TMT-based experiments were done in data-dependent acquisition mode with top 15 ions isolated at a window of *m/z* 0.7 and default charge state of +2. Precursors with charge states ranging from +2 to +7 were considered for MS/MS events. Normalized stepped collision energy was applied to fragment precursors at energies of 15%, 25%, and 40% for glycoproteomics and of 34% for proteomics. Precursor ions were acquired in the Orbitrap mass analyzer in *m/z* range of 350–2,000 for glycoproteomics and *m/z* 350–1,800 for proteomics at a resolution of 120,000 (at *m/z* 200). Automatic gain control (AGC) for MS and MS/MS were 10^6^ and 1 × 10^5^, and injection times to reach AGC were 50 ms and 250 ms, respectively. Exclude isotopes feature was set to “on,” and 60-second dynamic exclusion was applied. Data acquisition was performed with option of “lock mass” (*m/z* 441.12002) for all data.

MAX-enriched glycopeptides were analyzed on an Orbitrap Eclipse mass spectrometer connected to UltiMate 3000 liquid chromatography system. Glycopeptides were trapped on a trap column (100 mm × 2 cm, Acclaim PepMap100 Nano-Trap) at a flow rate of 20 μL/min. LC separation was performed on an analytical column (EASY-Spray 75 μm × 50 cm, C_18_ 2 μm, 100 Å) with a flow rate of 300 nL/min with a linear gradient of solvent B (100% ACN, 0.1% formic acid) over a 150-minute gradient. All label-free experiments were done in data-dependent acquisition mode at an isolation window of *m/z* 1.6 and default charge state of +2. Precursor ions were acquired at a resolution of 120,000 (at *m/z* 200) and at a resolution of 30,000 (at *m/z* 200) for fragment ions. Precursor fragmentation was carried out using normalized stepped higher-energy collisional dissociation at 15%, 25%, and 40%. The scans were acquired in top-speed method with 3-second cycle time between MS and MS/MS.

### Targeted LC-MS/MS analysis

The targeted LC-MS/MS analysis was carried out on an Orbitrap Eclipse mass spectrometer connected to UltiMate 3000 RSLC nano (Thermo Fisher Scientific). MAX-enriched serum glycopeptide samples were injected into trapping column (PepMap C18, 2 cm × 100 μm, Thermo Fisher Scientific) at the flow rate of 10 μL/min. After sample loading, samples were transferred to analytical column (PepMap RSLC C_18_ 2 μm, 75 μm × 50 cm, Thermo Fisher Scientific) at the flow rate of 300 nL/min. The analytical gradient started at 3% mobile phase B, and then mobile phase B was increased to 40% over 24 minutes, increased to 95% over 2 minutes, and maintained at 95% for another 7 minutes. Thereafter, mobile phase B was decreased to 2%, and the analytical column was reconditioned for 7 minutes. While an electrospray voltage of 2.0 kV was fixed in positive ion mode, MS spectra at a resolution of 120,000 and MS/MS spectra at a resolution of 30,000 in PRM mode were obtained with stepped collision energy set at 15%, 30%, and 45%. Skyline was used to build the inclusion list, which incorporated the targeted precursor *m/z* of glycopeptides and charge state. All targeted PRM raw files were processed in Skyline to generate extracted ion chromatogram and perform peak area integration (58).

### Database searching and data analysis

Database searching was performed using publicly available software pGlyco Version 2.2.0; pGlyco version 3.0 was used to search for glycosylation at multiple glycosylation sites (37, 59). Glycan databases already available with the software were used, and Uniprot Human Reviewed protein sequences (20,432 entries, downloaded February 1, 2021) were used as protein sequence FASTA file. Fully tryptic cleavage specificity with 2 missed cleavages was used, and precursor and fragment tolerance were set to 10 and 20 ppm, respectively. Carbamidomethylation of cysteine was set as fixed modification and oxidation of methionine as a variable modification. For TMT-based experiments, TMTPro at lysine was set as a fixed modification, and TMTPro at peptide N-terminal was set as variable modification. The results were filtered to retain entries that had 5% FDR at glycopeptide level. Reporter ion quantitation was performed on Proteome Discoverer 2.5 (reporter ion quantifier node), and IDs were matched with quantitation on a scan-to-scan (MS/MS) basis. Glycopeptide-spectrum matches were combined to reflect only unique glycopeptides per search by glycan composition, and peptide sequence and summed-up reporter ion intensities were calculated. Spectra were manually verified for several glycan oxonium ions and quality. All sialic acid glycopeptides’ spectra were verified for presence of sialic acid–specific oxonium ions at *m/z* 274.09, 292.10 and/or 657.23. Multiply glycosylated glycopeptides were verified for evidence of glycosylation (peptide b or y ion+glycan fragment) at both sites. The proteomics data set was searched using Sequest in Proteome Discoverer 2.5.

### Statistics

Fold-changes of glycopeptides, peptides, and proteins were calculated as average values from affected individuals over average values from control samples. The *P* values were obtained using unpaired 2-tailed *t* test. The *P* values were adjusted (reported *q* values) by Benjamini-Hochberg procedure for multiple comparisons on Perseus, and publicly available MetaboAnalyst 5.0 was used to draw PCA score plots (60, 61). A *q* value less than 0.05 was considered significant. Perseus was used to draw heatmaps.

Levels of Man_5_GlcNAc_2_ glycopeptide from C4 at site Asn^226^ were analyzed to determine if they correlated with the NPCRS scores of the affected individuals. Given the differences in protein expression data measurement methodology across study phases along with the ordinal nature of the NPCRS scores, we considered a meta-analytic strategy in which individual nonparametric rank correlation testing was performed for data within a given phase, and the results were combined using *P* value combination approaches. Specifically, Kendall’s Tau rank correlation test was performed to test the null hypothesis of zero correlation under a 2-sided alternative, with *P* values derived using the normal approximation with continuity correction. The *P* values resulting from testing across the 3 phases were then combined using a modified form of the Stouffer’s weighted Z approach. Weights were defined based on the square root of the case sample size of the corresponding phases. To account for directionality, sign of the corresponding tau estimate was applied to the weighting in the calculation of the numerator of the *z*-score (e.g., moderate evidence that is consistently in the same direction will have a compounding effect on the result). For analyses of individual scoring items, similar rank correlation testing was performed as described above. Given the small number of participants per study phase, there is elevated risk of lack of variation for the score item values, preventing hypothesis testing. Under these conditions, usage of Stouffer’s combined Z approach was only considered if at least 2 study phases were able to have hypothesis testing performed.

### Study approval

All affected individuals included in this work are enrolled in the FCDGC natural history study (Mayo Clinic IRB 19-005187; https://clinicaltrials.gov/ct2/show/NCT04199000?cond=C-DG&draw=2&rank=4). Written informed consent was obtained from the legally authorized representatives of the individuals prior to study initiation. Control serum samples were used from unaffected individuals who donated deidentified residual samples (IRB: 21-012890).

## Supplementary Material

Supplementary file

Supplementary data values

Supplementary table 1

Supplementary table 2

Supplementary table 3

Supplementary table 4

## Figures and Tables

**Table 1:** Salient features of affected individuals

Feature	Number of affected individuals
Total	35
Males	22
Females	13
**Age (y)**	
Median	10
Range	1–36
**Clinical severity (NPCRS) score**	
Median	23
Range	9–37
**Affected individuals by NPCRS score**	
Mild (0–14 points)	6
Moderate (15–25 points)	18
Severe (>26 points)	11
**Commonest pathogenic variants in ** ***PMM2***	
p.R141H	20
p.T237R	5
p.F119L	4
p.C241S	4
p.P113L	4

NPCRS, Nijmegen Progression CDG Rating Scale.

**Table 2:** Affected individuals and their clinical features

Analysis set	Sample ID	Age (y)	Sex	NPCRS^A^				Neurologic symptoms		Other unique features and clinical remarks
				Section I	Section II	Section III	Total	Ataxia with GDD^B^	Seizures	
Discovery set	Sample 1	6	M	15	5	16	36	+	-	Stroke-like episodes
	Sample 2	6	M	11	4	14	29	+	-	
	Sample 3	6	F	11	0	13	24	+	-	Epalrestat trial (pretreatment)
	Sample 4	7	F	9	3	12	24	+	-	
	Sample 5	5	M	7	1	10	18	+	-	Absence of neuropathy
	Sample 6	15	M	6	5	9	20	+	+	
	Sample 7	7	M	12	3	13	28	+	+	Congenital heart defect
Profiling set	Sample 8	2	M	7	3	8	18	+	-	Absence of neuropathy
	Sample 9	3	M	15	4	18	37	+	-	Chest deformity
	Sample 10	36	F	6	4	13	23	+	-	Able to walk
	Sample 11	28	M	11	6	15	32	+	+	Recurrent thrombotic episodes
	Sample 12	7	M	17	3	14	34	+	-	Jejunal tube feeding/hearing loss
	Sample 13	4	F	9	4	13	26	+	+	
	Sample 14	24	M	9	1	14	24	+	-	
	Sample 15	7	F	6	0	12	18	+	-	Epalrestat trial (on treatment)
	Sample 16	14	M	9	2	12	23	+	-	Scoliosis
	Sample 17	1	M	8	2	9	19	+	-	Absence of neuropathy
	Sample 18	22	M	13	8	15	36	+	+	
	Sample 19	31	M	3	0	9	12	+	+	Absence of neuropathy
	Sample 20	21	F	5	4	9	18	+, #	-	
	Sample 21	13	M	6	4	11	21	+	-	
	Sample 22	11	F	5	1	4	10	+, #	-	Absence of neuropathy/able to walk
	Sample 23	10	F	9	1	12	22	+	-	Absence of neuropathy/able to walk
	Sample 24	6	F	15	2	13	30	+	-	After liver transplant
	Sample 25	12	M	4	0	10	14	+	-	Able to walk
	Sample 26	23	F	3	3	3	9	+, #	-	Absence of neuropathy/able to walk
	Sample 27	11	F	9	4	13	26	+	+	Hearing loss/retinitis pigmentosa
	Sample 28	9	M	10	2	14	26	+	+	Hearing loss/retinitis pigmentosa
Blinded set	Sample 29	7	M	9	2	13	24	+	-	
	Sample 30	25	F	3	3	3	9	+, #	-	Absence of neuropathy/able to walk
	Sample 31	9	M	6	1	10	17	+	-	
	Sample 32	4	M	4	1	7	12	+	-	Absence of neuropathy
	Sample 33	9	F	6	1	11	18	+	-	
	Sample 34	11	M	5	1	9	15	+	-	
	Sample 35	10	M	7	3	13	23	+	+	
	Sample 36	16	F	5	5	7	17	+	-	Absence of neuropathy/able to walk

List of included individuals with PMM2-CDG with demographic information and clinical findings.

A Nijmegen Progression CDG Rating Scale. Patients are scored on 3 sections as follows: section I (current function, 21 points), section II (system-specific involvement, 30 points), and section III (current clinical assessment, 31 points) for a total score of 82 points; using these scores, cases are placed into mild (0–14 points), moderate (15–25 points), or severe (>26 points) categories.

B Global developmental delay. +, present; -, absent; #, learning disability without intellectual disability.

**Table 3:** Affected individuals and their laboratory features

Analysis set	Sample ID	Genetic variants in *PMM2* (predicted protein level, allele 1/allele 2)	Abnormal glycosylated coagulation factors and hormones	CDT by intact mass spectrometry		Result of glycopeptide analysis (Complement C4-derived glycopeptide bearing Man_5_HexNAc_2_ at Asn^226^)
				Mono- to di-oligo ratio (normal [N] ≤ 0.06)	A- to di-oligo ratio (normal [N] ≤ 0.011)	
Discovery set	Sample 1	R141H/F119L	+	0.14 (↑)	0.005	Detected
	Sample 2	P113L/D148N	+	0.054 (N)	0.123 (↑)	Detected
	Sample 3	R141H/E139K	-	0.09 (↑)	0.004 (N)	Detected
	Sample 4	D188G/V231M	+	0.3 (↑)	0.31 (↑)	Detected
	Sample 5	R141H/F183S	+	0.69 (↑)	0.196 (↑)	Detected
	Sample 6	R141H/I153T	+	0.85 (↑)	0.367 (↑)	Detected
	Sample 7	R69C/D148N	-	0.12 (↑)	0.007 (N)	Detected
Profiling set	Sample 8	Q37X/P113T	+	0.51 (↑)	0.031 (↑)	Detected
	Sample 9	R141H/V129M	+	0.58 (↑)	0.112 (↑)	Detected
	Sample 10	F119L/F119L	+	1.06 (↑)	0.520 (↑)	Detected
	Sample 11	R141H/F119L	+	1.31 (↑)	0.656 (↑)	Detected
	Sample 12	R141H/F119L	+	0.14 (↑)	0.005 (N)	Detected
	Sample 13	R141H/V129M	+	0.70 (↑)	0.314 (↑)	Detected
	Sample 14	C9Y/D148N	+	0.46 (↑)	0.147 (↑)	Detected
	Sample 15	R141H/E139K	-	0.09 (↑)	0.004 (N)	Detected
	Sample 16	R141H/I132T	+	0.75 (↑)	0.212 (↑)	Detected
	Sample 17	R141H/F68C	+	0.61 (↑)	0.190 (↑)	Detected
	Sample 18	P113L/-	+	0.791 (↑)	0.244 (↑)	Detected
	Sample 19	R141H/P113L	-	0.81 (↑)	0.60 (↑)	Detected
	Sample 20	R141H/C241S	+	0.8133 (↑)	0.0639 (↑)	Detected
	Sample 21	A108V/T237R	+	0.42 (↑)	0.097 (↑)	Detected
	Sample 22	Y229S/T237R	+	0.79 (↑)	0.376 (↑)	Detected
	Sample 23	Y229S/T237R	+	1.20 (↑)	0.628 (↑)	Detected
	Sample 24	P113L/T237R	-	0.04 (N)	0.004 (N)	Detected
	Sample 25	R141H/C241S	-	0.06 (N)	0.004 (N)	Detected
	Sample 26	R123Q/C241S	+	0.05 (N)	0.004 (N)	Detected
	Sample 27	R141H/-	+	0.03 (N)	0.002 (N)	Detected
	Sample 28	R141H/-	-	0.04 (N)	0.003 (N)	Detected
Blinded set	Sample 29	R141H/T118A	-	0.36 (↑)	0.064 (↑)	Detected
	Sample 30	R123Q/C241S	+	0.08 (↑)	0.013 (↑)	Detected
	Sample 31	R141H/I153T	+	0.67 (↑)	0.176 (↑)	Detected
	Sample 32	R141H/A108V	+	0.1069 (↑)	0.0607 (↑)	Detected
	Sample 33	R141H/G186R	-	0.2 (↑)	0.017(↑)	Detected
	Sample 34	A108V/T237R	-	0.18 (↑)	0.025 (↑)	Detected
	Sample 35	Q33P/S47L	+	0.54 (↑)	0.161 (↑)	Detected
	Sample 36	R141H/Y229S	+	1.9322 (↑)	0.5024 (↑)	Detected

List of included individuals with PMM2-CDG with genetic information and biochemical findings, including results of intact mass analysis testing for carbohydrate-deficient transferrin (CDT) and results of glycopeptide analysis for Man_5_GlcNAc_2_ glycopeptide from complement C4 at Asn^226^. Participants corresponding to 3 samples had noncoding pathogenic variants in their second allele: sample 18, IVS3+2T>C; samples 27 and 28, c.640-23A>G.

## Data Availability

The underlying values for data presented in the graphs and supplemental figures are available in the Supporting Data Values file. The MS proteomics data have been deposited to the ProteomeXchange Consortium via the PRIDE (62) partner repository with the data set identifier PXD042446.
