# Supplementary material for: A complement C4–derived glycopeptide is a biomarker for PMM2-CDG
Source: JCI Insight. Author manuscript; Available in PMC 2024 May 3. (PMC7615924; doi:10.1172/jci.insight.172509)
Supplement: Supplementary file [file EMS195519-supplement-Supplementary_file.pdf]

Supplemental Figures 1-3 for

A complement C4-derived glycopeptide as a biomarker for PMM2-CDG

Kishore Garapati[#], Rohit Budhraja[#], Mayank Saraswat, Jinyong Kim, Neha Joshi, Gunveen S. Sachdeva, Anu Jain, Anna N. Ligezka, Silvia Radenkovic, Madan Gopal Ramarajan, Savita Udainiya, Kimiyo Raymond, Miao He, Christina Lam, Austin Larson, Andrew C. Edmondson, Kyriakie Sarafoglou, Nicholas B. Larson, Hudson H. Freeze, Matthew J. Schultz, Tamas Kozicz, Eva Morava[*], Akhilesh Pandey[*]

[#]Kishore Garapati and Rohit Budhraja are co-first authors

***Corresponding authors**

Akhilesh Pandey, M.D., Ph.D.
Department of Laboratory Medicine and Pathology
Mayo Clinic
200 First Street SW
Rochester, MN 55905, USA
Tel: +1-507-293-9564
Email: pandey.akhilesh@mayo.edu

Eva Morava, M.D., Ph.D.
Department of Genomics and Genetic Sciences
Ichan School of Medicine at Mount Sinai Hospital
New York, NY 10029, USA
Tel: +1- 212-659-6841
Email: eva.morava@mssm.edu

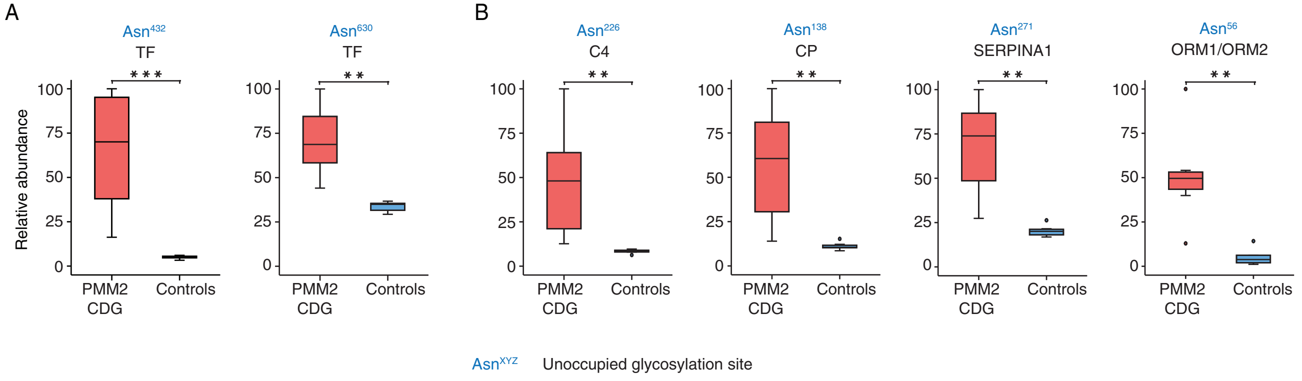

Supplemental Figure 1. Absent glycosylation. Box plots showing increased levels of unoccupied glycosylation sites on proteins (A) Transferrin-derived peptides with non-glycosylated sites Asn432 and Asn630 (B) Peptides derived from other selected proteins with unoccupied glycosylation sites as indicated. The box plots depict minimum and maximum values (whiskers), upper and lower quartiles, and median. The length of the box represents the interquartile range. PMM2-CDG (n=7), Controls (n=7); *=q<0.05, **=q<0.01, ***=q<0.001; The q values were calculated by t-test with multiple testing using Benjamini-Hochberg procedure.

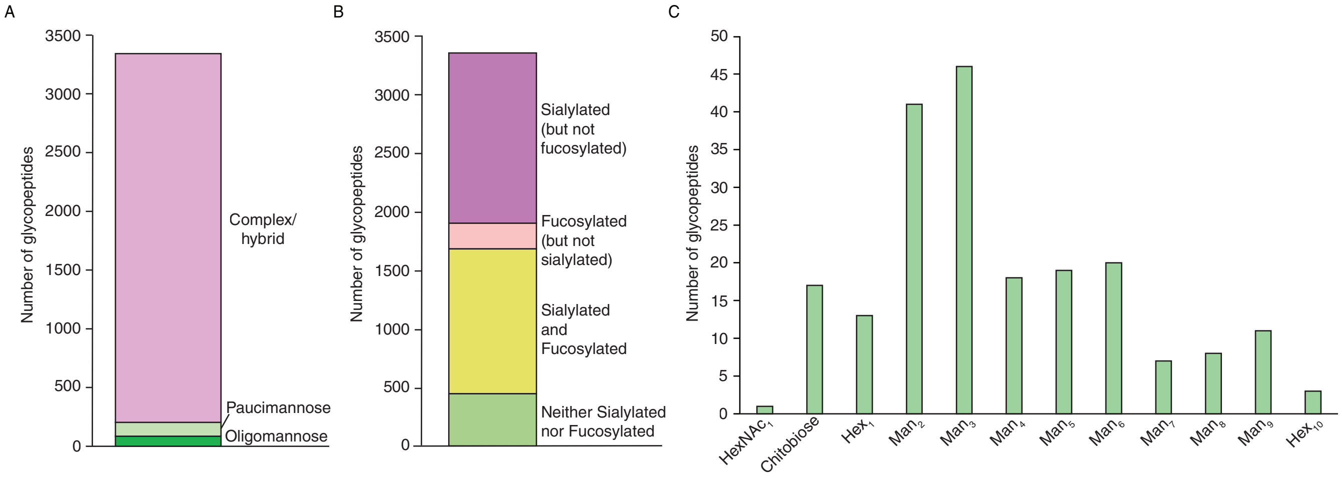

Supplemental Figure 2. Serum glycoproteomics. (A) Bar plot showing numbers of glycopeptides by class (oligomannose, paucimannose, complex/hybrid) (B) Bar plot showing numbers of glycopeptides by composition (sialylated, fucosylated, sialylated and fucosylated, neither sialylated nor fucosylated) (C) Number of paucimannose and oligomannose glycopeptides containing different numbers of hexose residues.

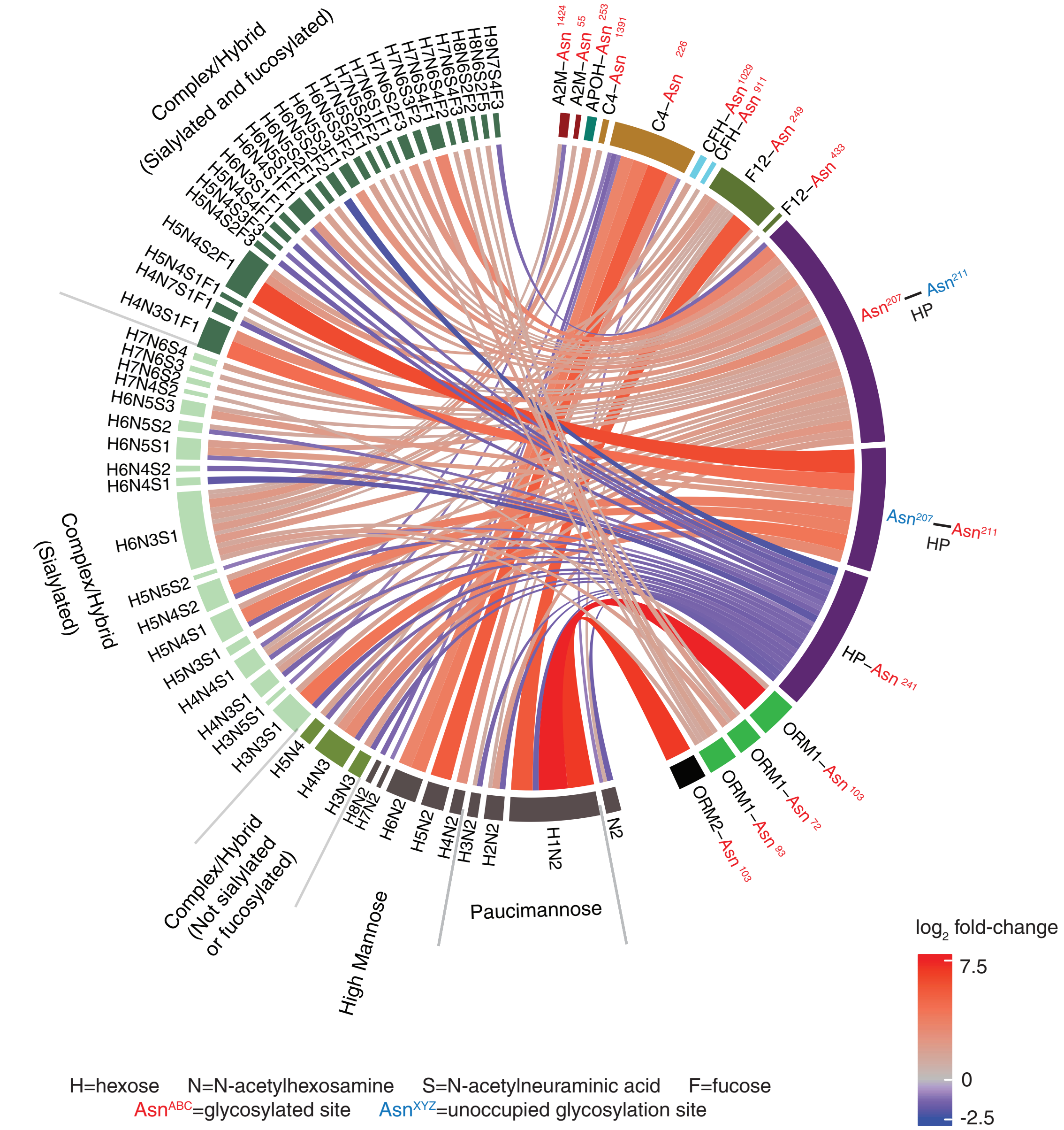

Supplemental Figure 3. Site-specific changes in glycosylation of selected abundant proteins. Data are shown as a chord diagram with each connecting chord representing a glycopeptide from a protein glycosylation site shown on one side, and the composition of the glycan on the other side. The color of the chord represents the $\log_2$-transformed fold-change (average of PMM2-CDG, n=7/average of controls, n=7) as shown.
